# Data of preparation and evaluation of supramolecular hydrogel based on cellulose for sustained release of therapeutic substances with antimicrobial and wound healing properties

**DOI:** 10.1016/j.dib.2020.105902

**Published:** 2020-06-21

**Authors:** Adolfo Marican, Oscar Forero-Doria, Efrain Polo, Jaime Gallego, Esteban F. Durán-Lara

**Affiliations:** a Instituto de Química de Recursos Naturales, Universidad de Talca, Talca 3460000, Maule, Chile; b Química de Recursos Energéticos y Medio Ambiente, Instituto de Química, Facultad de Ciencias Exactas y Naturales, Universidad de Antioquia UdeA, Calle 70 No. 52-21, Medellín, Colombia; c Bio & NanoMaterials Lab, Drug Delivery and Controlled Release, Universidad de Talca, Talca 3460000, Maule, Chile; d Departamento de Microbiología, Facultad de Ciencias de la Salud, Universidad de Talca, Talca 3460000, Maule, Chile

**Keywords:** Hydrogels, Cellulose, Nanotube, Chalcone, Sustained release, Swelling degree, Thermogravimetric analysis

## Abstract

The data article refers to the paper “supramolecular hydrogel based on cellulose for sustained release of therapeutic substances with antimicrobial and wound healing properties”[1]. The dataset includes the synthesis and characterization of (E)-1,3-bis(4-(allyloxy)phenyl)prop‑2-en-1-one (3) (crosslinking agent). Moreover, the multiwall carbon nanotubes (MWCNTs) synthesis and functionalization (MWCNTs-COOH) are described. The formulation obtained by adding multiwalled carbon nanotubes-COOH with the crosslinked cellulose-chalcone hydrogel is abbreviated as MWCNTsCCH, and the same formulation loaded with therapeutic substances (TS) is named MWCNTsCCH-TS. The MWCNTsCCH database such as components and their amounts, swelling degree, thermogravimetric analysis, and cytotoxicity evaluation are depicted. Finally, to elucidate the mechanism of therapeutic substances release, the obtained averages of the release profiles were fitted through mathematical models.

**Table:** 

**Subject**	Organic Chemistry
**Specific subject area**	Preparation, characterization and evaluation of supramolecular hydrogel based on cellulose
**Type of data**	Table
	Image
	Graph
	Figure
	Filtered data
	Raw data
**How data were acquired**	**Mass spectrometry experiments**. These analyses were carried out on a high-resolution high accuracy hybrid double quadrupole (Qq) and orthogonal time-of-flight (Tof) mass spectrometer (QTof, Micromass UK). The temperature of the nebulizer was 50 °C. The ESI source and the mass spectrometer were operated in the positive-ion mode.
	**FT-IR:** Fourier transform infrared spectroscopy (NEXUS 670 FT-IR, Thermo Nicolet, Madison, WI, USA). Samples for FT-IR measurements were prepared by grinding dry material into KBr in an agate mortar at a very low concentration of compound 3 (0.03 wt%). The wavenumber range scanned was 4000–400 cm^−1^; 32 scans of 2 cm^−1^ resolution were signal-averaged and stored.
	**NMR**. The ^1^H NMR and ^13^C NMR spectra were recorded on a Bruker (400 MHz) spectrometer. Chemical shifts (δ) are recorded in ppm with the solvent resonance as the internal standard and coupling constants (J) recorded in Hz.
	**Equilibrium swelling ratio of MWCNTsCCH:** The water uptake activity was calculated by the equilibrium swelling ratio (% ESR). The ESR of the hydrogel samples was estimated according to the following equation:
	ESR (%)$=\frac{W_{w}-W_{d}}{W_{d}}\;x\;100\%$
	**TGA**: thermogravimetric analyzer STD 650 TA-235 instruments by TA-instruments. ≈ 3 mg of freeze-dried sample was placed into the balance and were heated to a constant heating rate of 10 °C min^−1^. The heating was realized from room temperature to 800 °C in N_2_ or air as a reactive gas (with a mass flow of 50 mL min^−1^.), which was switched when the temperature reached 800 °C, and hold at this temperature during 30 min, for allowing the oxidation process. Also, 50 mL min^−1^ of N_2_ was used as protection gas into the electronic balance; around 3 mg of the composite was placed into a Pt crucible for each analysis.
	**HPLC:** Perkin Elmer series 200 HPLC system (Norwalk, CT, USA) with a UV–vis detector. A YWG C-8 (250 mm x 4.6 mm i.d. x 10 μm) column was utilized for the sample analysis.
	**Cytotoxicity and cell viability**. The data were acquired from fibroblast MTT assay.
**Data format**	Raw data of models: Microsoft Excel; GraphPad Prism 8
**Parameters for data collection**	According to the respective experimental measurements, some data were acquired at specific intervals during a period of time and other data were collected at a specific pH and temperature.
**Description of data collection**	The data for structural characterization of compound 3 was obtained by ^1^H–^13^C NMR, FT-IR and time-of-flight mass spectrometry analysis.
	The data of formulation components and their amounts were obtained according to the encapsulation methodology performed.
	The data of equilibrium swelling ratio (% ESR) were collected measuring the water uptake activity of hydrogel at specific time intervals.
	The in vitro kinetic releases of the therapeutic substances (TSs) from MWCNTsCCH-TS were obtained under physiological conditions (33.5 °C, PBS at pH 7.4). The samples were assessed by HPLC. The cumulative released percent of each TS was monitored over time.
	The data of viability studies of MWCNTsCCH were obtained evaluating the fibroblast cell viability after exposure to MWCNTsCCH.
**Data source location**	Institution: Universidad de Talca
	City/Town/Region: Talca, Maule
	Country: Chile
**Data accessibility**	Data is provided with this article
**Related research article**	Oscar Forero-Doria, Efrain Polo, Adolfo Marican, Luis Guzmán, Bernardo Venegas, Sekar Vijayakumar; Sergio Wehinger, Marcelo Guerrero, Jaime Gallego and Esteban F. Durán-Lara, Supramolecular hydrogel based on cellulose for sustained-release of therapeutic substances with antimicrobial and wound healing properties, Carbohydrate Polymers, 242 (2020) 116,383. https://doi.org/10.1016/j.carbpol.2020.116383.

**Value of the Data**

The data presented is useful to know the behavior release if therapeutic substances from carbon nanotube-containing cellulose-chalcone hydrogels, with the goal of achieving multiple therapeutic effects.

The beneficiaries of these data are researchers who investigate the release of multiple drugs from polymeric matrices of cellulose-based hydrogels.

The data presented will allow the development of new polymeric platforms of cellulose-based hydrogels, being a guide for the determination of the behavior of these compounds for the release of multiple drugs.

## 1 Data description

**Fig. 1:**
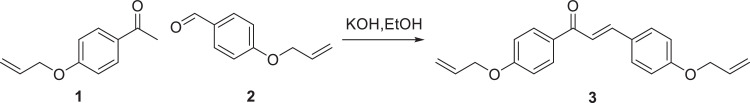
Synthesis of (E)−1,3-bis(4-(allyloxy)phenyl)prop‑2-en-1-one (3).

**Fig. 2:**
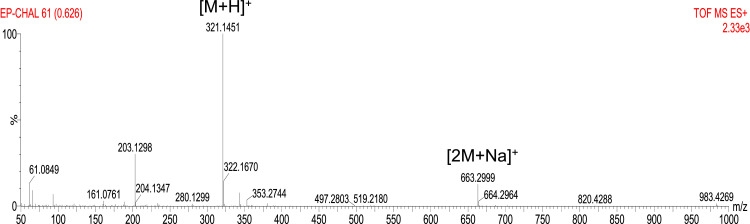
Mass spectrum (TOF MS ES+) of **3.**

**Fig. 3:**
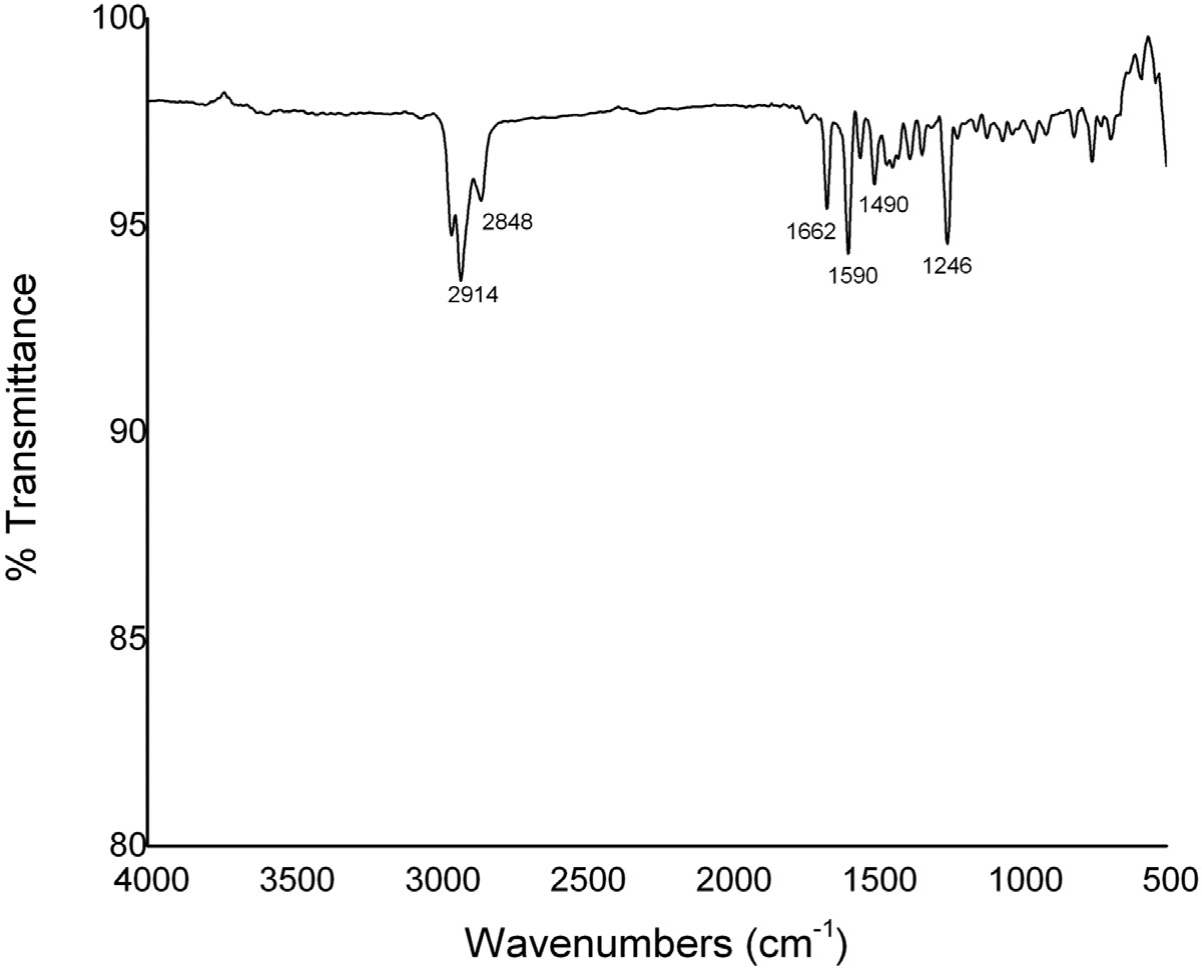
FTIR spectra of Chalcone 3.

**Fig. 4:**
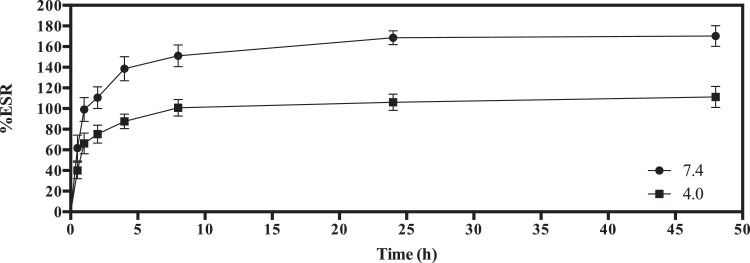
The swelling ratio of the MWCNTsCCH at 24 °C as a function of time and pH. Data are shown as mean ± SD (*n* = 3).

**Fig. 5:**
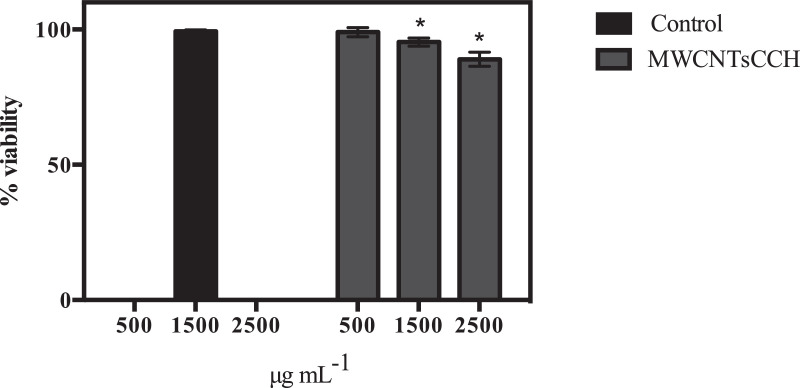
Percentage of cell viability obtained from the MTT assay of the L929 fibroblast cells with respect to negative control (without MWCNTsCCH). Each bar indicates mean ± relative standard deviations (RSD) of three replications. Bars not labeled by the same letter represent statistical significance at *P* ≤ 0.05 using ANOVA followed by Tukey's HSD test.

The synthesis of crosslinker (E)−1,3-bis(4-(allyloxy)phenyl)prop‑2-en-1-one (3) is represented in Fig. 1 and detailed description of the reaction procedure is stated in the experimental design, materials, and methods section. The compound **3** was characterized by TOF mass and FTIR spectroscopy analysis (see Figs. 2 and 3). The preparation of MWCNTsCCH and loading of therapeutic substances (MWCNTsCCH-TS) are depicted in Table 1 and detailed synthesis is explained in experimental design, materials, and methods section. Moreover, we have depicted the loaded therapeutic substances, and their amounts according to the standard concentrations of bioactive compounds applied in dermatology. The Equilibrium Swelling Ratio (ESR) of MWCNTsCCH was evaluated at pH values of 7.4 and 4.0 (see Fig. 4 and Table 2). Graphs of the study results were designed by utilizing GraphPad Prism 6. Statistical significance was set at *p* < 0.05. The mass loss (TG) and derivative (DTG) curves were obtained in the interval of room temperature to 800 °C, this data are showed in Table 3. The release percentage of linezolid, allantoin, dexpanthenol, and resveratrol from MWCNTsCCH are shown in Table 4. These data allow obtain the release profile and to calculate release kinetic of TS. To elucidate the mechanism of TS release, the data of Table 5 were obtained by applying different mathematical modeling drug-release equations, namely, zero-order (Eq. (3)), first-order (Eq. (4)), Hixson-Crowell (Eq. (5)), Higuchi (Eq. (6)), Korsmeyer–Peppas (Eq. (7)), and Peppas-Sahlin (Eq. (8)) equations [2], [3]. Fig. 5 and Table 6 demonstrates the CNTs-CCH cytotoxicity. This experiment was carried out to measure fibroblast cell viability after exposure to MWCNTsCCH. The biocompatibility of sterilized MWCNTsCCH after 24 h was evaluated by a cell viability assay using L929 fibroblast cells. Fig. 5 shows the fibroblast cell viability in the presence of three different concentrations of MWCNTsCCH (500, 1500, and 2500 μg mL^−1^)**.**

**Table 1:** Formulation components and their amounts.

Hydrogel	Crosslinker	% Crosslinker ratio	% MWCNTs	Therapeutic Substances (%)			
				Allantoin	Resveratrol	Dexpanthenol	Linezolid
MWCNTsCCH-TS	Chalcone	20	15	5	2	2	2

The weight percentage of Chalcone, MWCNTs, and TSs is in relation to the cellulose mass.

**Table 2:** The swelling ratio data of the MWCNTsCCH at 24 °C as a function of time and pH.

Time[h]	pH			
	7.4		4.0	
	%ESR	%RSD	%ESR	%RSD
0	0	0	0	0
0.5	61.81	8.04	40.10	4.51
1	99.03	6.05	66.24	5.50
2	110.54	6.02	75.25	4.11
4	138.59	5.49	87.62	3.39
8	151.08	6.56	100.72	4.95
24	168.57	4.92	106.04	2.67
48	170.23	5.51	111.27	4.49

(*) RSD: relative standard deviations.

**Table 3:** TG and DTG analysis of principal steps of mass loss of MWCNTs-COOH and MWCNTsCCH at a heating rate of 10 °C min^−1^ in nitrogen until 800 °C, at this temperature the gas was switched to air flow and holding the temperature (at 800 °C) during 15 min.

Material	Steps	*T*_i_/ °C	*T*_m_/ °C	*T*_f_/ °C	Mass loss /%	Conditions
MWCNTs-COOH	**I**	30	74	169	29.0	N_2_ at 10 °C min^−1^
	**II**	169	234	302	46.9	
	**III**	302	550	800	0	
	**IV**	800			83.0	Isothermal at 800 °C under air
MWCNTsCCH	**I**	57	119	172	6.7	N_2_ at 10 °C min^−1^
	**II**	172	211	223	11.9	
	**III**	223	301	359	62.6	
	**IV**	359	439	558	74.2	
	**V**	558	690	800	80.1	
	**VI**	800			100	Isothermal at 800 °C under air
Initiation temperature, *T*_i_/ °C; Maximum mass loss temperature, *T*_m_/ °C; Final temperature, *T*_f_/ °C

**Table 4:** TS release percentage at different times (ND: Not Detected).

Time[h]	Linezolid[R*elease*% ± RSD](*n* = 3)	Allantoin[R*elease*% ± RSD](*n* = 3)	Dexpanthenol[R*elease*% ± RSD](*n* = 3)	Resveratrol[R*elease*% ± RSD](*n* = 3)
0	ND	ND	ND	ND
1	9.57 ± 2.56	12.29 ± 2.23	18.32 ± 2.32	20.95 ± 5.35
2	17.56 ± 3.21	22.78 ± 2.13	35.64 ± 3.16	41.55 ± 1.34
4	20.71 ± 3.31	30.49 ± 2.65	44.23 ± 3.72	51.00 ± 3.82
6	24.59 ± 4.59	38.07 ± 1.97	51.67 ± 1.16	56.00 ± 2.00
12	30.08 ± 9.29	43.80 ± 3.18	55.60 ± 2.31	61.00 ± 3.61
24	34.81 ± 5.69	51.80 ± 2.84	63.13 ± 3.70	70.33 ± 3.51
48	37.30 ± 3.76	57.73 ± 2.72	71.13 ± 3.40	77.00 ± 2.00
72	41.52 ± 7.42	64.00 ± 3.60	76.20 ± 2.27	82.67 ± 4.51
96	44.49 ± 5.16	68.80 ± 1.11	80.87 ± 2.49	87.00 ± 4.58
120	47.92 ± 7.52	72.80 ± 2.03	84.33 ± 2.52	90.67 ± 3.06

**Table 5:** TS release kinetics and correlation coefficient values from Fick, Hixon-Crowell, Higushi and Korsmeyer-Peppas models.

TS	Model										
	Zero Order		First Order		Hixon-Crowell		Higushi		Korsmeyer-Peppas		
	R^2^	K	R^2^	K	R^2^	K	R^2^	K	R^2^	K	n
Linezolid	0.77757	0.48624	0.71528	0.03668	0.27698	−0.01421	0.86995	1.86614	0.90636	5.770985724	0.38125
Allantoin	0.81560	0.72056	0.86509	0.03073	0.30320	−0.01709	0.88539	1.18399	0.90133	3.02113408	0.43296
Dexpanthenol	0.72147	0.90251	0.66389	0.04393	0.23162	−0.01629	0.81194	3.19605	0.80952	11.84213479	0.35559
Resveratrol	0.70428	0.97783	0.65425	0.04489	0.21297	−0.01623	0.78669	3.36034	0.78726	13.67634349	0.33954

**Table 6:** Percentage data of cell viability obtained from the MTT assay.

[μg mL^−1^]	Control		MWCNTsCCH	
	%Viability	%RSD	%Viability	%RSD
500	–	–	99.0	1.73
1500	99.3	0.580	95.3	1.53
2500	–	–	89.0	2.65

During the isothermal oxidation at 800 °C under air atmosphere, the consumption of the high graphitized carbon takes place. These results show how the functionalization of the MWCNTs-COOH surface affects this graphitization process and therefore its oxidation resistance.

## 2 Experimental design, materials, and methods

### 2.1 Chalcone preparation (compound **3**)

The Chalcone was prepared according to the following method. A dry round-bottom flask (100 mL) was charged with p-hydroxyacetophenone and p-hydroxybenzaldehyde (20 mmol, 100 mol%), K_2_CO_3_ (40 mmol, 200 mol%), MeCN (20 mL), and allyl bromide (2.1 mL, 24 mmol, 120 mol%) under air at room temperature. The reaction mixture was stirred for 6 h at 60 °C. The resulting mixture was then extracted with EtOAc (100 mL). The organic layer was washed with saturated NH_4_Cl solution (2 × 60 mL), and then dried over Na_2_SO_4_, and subsequently concentrated in vacuo. This crude material might be used without further purification for the next steps. Next, 1-(4-(allyloxy)phenyl)ethan-1-one (1) (20 mmol) and 4-(allyloxy)benzaldehyde (2) (20 mmol) were stirred using KOH in ethanol (20 mL) for 12 h at room temperature. After the reaction was completed, the precipitate was filtered, washed with cold ethanol and water and dried under vacuum to afford a yellow solid (3). The physical data are listed below: Yield: 93% (5.9 g); yellow pale solid; mp 80–82 °C (lit 77–78 °C) [4]; IR (KBr, cm^−1^): 3076, 2923, 2868, 1650, 1595, 1502, 1415, 1259, 1163, 984, 822, 657; 1H NMR (90 MHz, CD_3_Cl): δ_H_ = 4.77 (bs, 4H), 5.44–5.70 (m, 4H), 6.06–6.42 (m, 2H), 7.08–7.22 (m, 4H), 7.68–7.89 (m, 3H), 8.07–8.26 (m, 3H); HRMS (ESI, *m/z*): calcd for C_21_H_21_O_3_ [*M* + *H*]^+^ 321.1491, found 321.1451; calcd for C_42_H_40_O_6_Na [2M+Na]^+^ 663,2722, found 663,2999.

### 2.2 Multiwall carbon nanotubes (MWCNTs) synthesis and functionalization (MWCNTs-COOH)

MWCNT were obtained through an ethanol decomposition reaction process at 900 °C in presence of perovskite-like oxide (LaFeO_3_) as a catalyst precursor by following the previously reported study [5]. The catalyst was placed into a horizontal reactor after which temperature was raised at 10 °C•min^−1^ under N_2_ atmosphere until the desired reaction temperature. Then ethanol at a 50% of volumetric fraction was injected over 4 h. Obtained MWCNTs were treated with 65% HNO_3_ and 96% H_2_SO_4_ (3:1), for 15 min at 130 °C, by applying a power of 500 W in a Milestone MicroSYNTH microwave reactor. The product was then filtered using a 0.45 μm pore size mixed cellulose-ester filter (advantec MFS, Inc.) and washed with deionized water until neutral pH. Filtered MWCNTs-COOH were dried at 100 °C for 24 h. This MWCNTs-COOH was characterized through different analytical techniques such as exhibited below [6]. The MWCNTs-COOH was termed as MWCNTs.

### 2.3 Equilibrium swelling ratio of MWCNTsCCH

The water uptake activity was calculated by the equilibrium swelling ratio (% ESR) at specific time intervals, according to the protocol of Marican, et al. 2018 [7]. The MWCNTsCCH film was placed in phosphate-buffered saline (PBS) (pH 7.4) and acetate buffer (pH 4.0) at ∼25 °C for 48 h until swelling equilibrium was reached. The weight of the wet sample [W_w_ (g)] was measured after cautiously removing surface moisture with absorbent paper. The weight of the dried sample [W_d_ (g)] was obtained after freeze-drying the prepared hydrogel. The ESR of the hydrogel samples was estimated according to the following equation (Eq. (1)):

(1) $$\text{ESR}\;\left(\%\right)=\frac{W_{w}-W_{d}}{W_{d}}\;x\;100\%$$

### 2.4 Thermogravimetric analysis

The sample analyses of MWCNTs and MWCNTsCCH were performed in a thermogravimetric analyzer (STD 650 TA-235, TA Instruments). Approximately 3 mg of freeze-dried sample was placed into the instrument balance and heated at a constant heating rate of 10 °C min^−1^. The heating was conducted from room temperature to 800 °C in N_2_ or air as a reactive gas (with a mass flow of 50 mL min^−1^). The temperature was held at 800 °C for 30 min to allow the oxidation process to complete. Additionally, 50 mL min^−1^ N_2_ was used as the protection gas in the electronic balance. Approximately 3 mg of the composite was placed into a platinum crucible for each analysis. The first region of the thermal analysis, from room temperature to 800 °C under an N_2_ atmosphere, examines the thermolabile molecules or fragments that can be decomposed by simple heating of the samples, such as the functional groups over the carbon nanotubes. The second region, the oxidative process (under O_2_), aims to observe the sample's oxidative resistance under extreme conditions, reactive gas (dynamic air atmosphere) and high temperature (800 °C). For the second region, once the temperature reaches 800 °C, the gas through the sample was switched to air (50 mL min^−1^), maintaining during all time the protective atmosphere across the electronic balance (50 mL min^−1^ of N_2_) and keeping the temperature isothermally at 800 °C.

### 2.5 Preparation of MWCNTsCCH and loading of therapeutic substances

First, 1 g of the cellulose solution was mixed with chalcone (CH) (3) at 20 w/w%. The synthesis and characterization of CH are shown in this data article, Section 2.1 and Figs. 1, 2 and 3. The reaction mixture was continuously sonicated for 2 h (the temperature of the sonication bath ranged from 25 to 60 °C) according to the modified method from Cass, et al. 2010 [8]. Then, a yellow viscous liquid corresponding to the crosslinked cellulose-chalcone prehydrogel was obtained. Second, MWCNTs (15 w/w%, MWCNTs-COOH, average size (diameter × length): 20–80 nm × 10–15 μm) were mixed with the prehydrogel and sonicated for 2 h at 50 °C. Next, the homogenized black mixture (prehydrogel-MWCNTs) was dialyzed for 2 days. Then, the therapeutic substances (TSs) allantoin, dexpanthenol, resveratrol, and linezolid were added to the solution [9]. The resulting mixture was sonicated for 2 h at 50 °C until a homogenized solution was obtained. Finally, the mixture solution was placed in an oven at 45 °C overnight until the crosslinking was complete and the hydrogel film was formed. The final composition of the loaded hydrogel is depicted in Table 1.

### 2.6 Drug release kinetics from MWCNTsCCH-TS

The protocol was performed according to Forero-Doria, et al., 2020 [1]. A known mass of MWCNTsCCH-TS (400 mg of dry sample) was placed in a flask, and 5 mL of PBS (pH 7.4) was poured over the formulation as a release medium. The flask was moved to an orbital shaker incubator water bath (Farazteb, Iran) at 33.5 ± 0.1 °C (skin temperature) and shaken at 35 ± 2 rpm. After every time interval, the PBS was recovered and replaced with an equal volume to maintain sink conditions during all studies. The samples and controls were analyzed by a Perkin Elmer series 200 HPLC system (Norwalk, CT, USA) with a UV–Vis detector. A YWG C-8 (250 mm x 4.6 mm i.d. x 10 μm) column was utilized for the sample analysis. A volume of 20 μL of eluent was injected into the equipment. The mobile phase utilized contained 20 mM K_2_HPO_4_ (pH 6.0, H_3_PO_4_)/methanol (90:10, v/v) in isocratic mode at a flow rate of 1.0 mL min^−1^. The samples were monitored at 210 nm (allantoin and dexpanthenol) and 300 nm (resveratrol) by absorbance detection at 30 °C. For quantification of linezolid, a stock solution (3 mg/mL) was prepared in methanol and stored at −18 °C. Standard solutions of linezolid were prepared with PBS (pH 7.4) in the range of 0.01 mg L^−1^ to 50 mg L^−1^. The chromatographic system consisted of a Perkin Elmer series 200 HPLC system (Norwalk, CT, USA) with a UV–vis detector and a C-18 Kromasil 100–5-C18 (250 mm × 4.6 mm i.d. × 5 μm) column. Fifty microliters of the sample was injected into the HPLC apparatus. Isocratic elution with methanol/water (50:50, v/v) at a constant flow rate of 1.0 mL min^−1^ was used as the mobile phase. The analytical wavelength was 254 nm at room temperature. The release rate of MWCNTsCCH-TS was obtained by applying the concentration of released and loaded TSs to the following correlation (Eq. (2)):

(2) $$\text{Cumulative}\;\text{TS}\;\text{release}\;\left(\%\right)=\;\text{Cumulative}\;\text{amount}\;\text{of}\;\text{TS}\;\text{released}\;\times \frac{100}{\text{Initial}\;\text{amount}\;\text{of}\;\text{TS}}$$

Drug release kinetics were carried out by applying different mathematical modeling drug-release equations, namely, zero-order (Eq. (3)), first-order (Eq. (4)), Hixson-Crowell (Eq. (5)), Higuchi (Eq. (6)), Korsmeyer–Peppas (Eq. (7)), and Peppas-Sahlin (Eq. (8)) equations:

(3) $$Q_{t}/Q_{0}=K_{0}t$$

(4) $$\text{ln}Q_{t}/Q_{0}=K_{1}t,$$

where Q_t_ is the amount of drug released at time t, and Q_0_ is the original drug concentration in the hydrogel.

(5) $$C_{0}^{1/3}--C_{t}^{1/3}=\text{Kt},$$

where C_t_ is the amount of drug released in time *t*, C_0_ is the initial amount of drug in the tablet, and K is the rate constant.

(6) $$Q=Kt^{1/2},$$

where Q is the cumulative drug release, K is the Higuchi release constant, and t is the time

(7) $$\frac{M_{t}}{M}=Kt^{n},$$

where M_t_/M is the cumulative drug release, K is the release constant, *t* is the time, and *n* is the release exponent.

(8) $$\frac{M_{t}}{M\infty }=Kdt^{n}+Krt^{2n},$$

where M_t_ and M_∞_ are the absolute cumulative amounts of drug release at time t and at infinite time, respectively.

### 2.7 Cytotoxicity and cell viability

CNTs-CCH cytotoxicity was assessed in fibroblast cells. For this aim, fibroblast biocompatibility was studied through the MTT assay according to the method of Mossman et al., 1983 [10]. Briefly, fibroblasts were seeded in 24-well plates (5 μL, 1.6 × 10^4^ cells per well), and 150 μL of Dulbecco's modified Eagle's medium (DMEM)-high glucose medium was added and incubated for 24 h at 37 °C in 5% CO_2_. Next, the medium was replaced by 100 μL of new DMEM-high glucose per well containing three different concentrations of MWCNTsCCH-TS (500 μg mL^−1^, 1500 μg mL^−1^, and 2500 μg mL^−1^). A new medium without MWCNTsCCH-TS was utilized as a control. Cell viability was analyzed after 24 h by MTT tests. Specifically, 5 μL of MTT solution (3 mg mL^−1^ in PBS) and 50 µL of new medium were added to each sample and incubated for 4 h at 37 °C in the dark; formazan crystals were then dissolved in 100 µL of dimethyl sulfoxide (DMSO) and incubated for 18 h. The supernatant was analyzed at 570 nm (Spectrophotometer, Packard Bell, Meriden, CT, USA). Untreated cells were referenced as controls with 100% viability. Finally, the cytotoxicity of MWCNTsCCH-TS on fibroblast cells was expressed as the relative viability (%), which correlates with the number of viable cells compared with the negative cell control (100%).

## Declaration of Competing Interest

The authors declare that they have no known competing financial interests or personal relationships, which have, or could be perceived to have, influenced the work reported in this article.

## Appendix Supplementary materials

Image, application 1

Image, application 2

Image, application 3

Image, application 4
